# Comparative effects of long- and short-interval aerobic high-intensity interval training on glycemic control among individuals with type 2 diabetes: a network meta-analysis

**DOI:** 10.1186/s13102-026-01921-4

**Published:** 2026-07-27

**Authors:** Xuanming Hu, Meilin Wen, Shunfang Liu

**Affiliations:** 1 Sport Performance Research Institute New Zealand (SPRINZ), Auckland University of Technology (AUT), Rosedale, Auckland, New Zealand; 2 School of Physical Education and Sports Science, South China Normal University, Guangzhou, Guangdong 510006 China

**Keywords:** Type 2 diabetes, High-intensity interval training, Moderate-intensity continuous training, Network meta-analysis

## Abstract

**Objective:**

This study aimed to systematically compare and rank the relative efficacy of moderate-intensity continuous training (MICT), short-interval high-intensity interval training (HIIT-S), and long-interval HIIT (HIIT-L) on glycemic control among individuals with type 2 diabetes (T2D) using a network meta-analysis (NMA).

**Methods:**

Following the PRISMA-NMA guidelines, we searched PubMed, Embase, Cochrane Library, and Web of Science from database inception to October 24, 2025. Randomized controlled trials (RCTs) comparing the effects of HIIT, MICT, or non-exercise control (CON) on glycemic outcomes among individuals with T2D were included. Based on the intensity of training and durations of each high-intensity interval, aerobic HIIT was further classified into HIIT-S (≤ 60 s) and HIIT-L (≥ 2 min). The primary outcomes were glycated hemoglobin (HbA1c) and fasting plasma glucose (FPG). The homeostatic model assessment for insulin resistance (HOMA-IR) was included post hoc as an exploratory secondary outcome. A Bayesian NMA using random-effects models was implemented using the gemtc package in R. Effect sizes were expressed as mean differences (MDs) with 95% credible intervals (CrIs), and interventions were ranked through the Surface Under the Cumulative Ranking curve (SUCRA). This study was prospectively registered on PROSPERO (CRD420251175046).

**Results:**

In total, sixteen RCTs involving 744 participants with T2D were included. Among them, fifteen studies reported HbA1c, thirteen reported FPG, and seven reported HOMA-IR. For HbA1c, MICT (MD = − 0.47, 95% CrI: −0.77 to − 0.17), HIIT-S (MD = − 0.50, 95% CrI: −0.84 to − 0.18), and HIIT-L (MD = − 0.85, 95% CrI: −1.16 to − 0.55) all significantly reduced HbA1c levels relative to CON. Moreover, HIIT-L was significantly more effective than MICT (MD = − 0.38, 95% CrI: −0.76 to − 0.01), whereas no significant difference was observed between HIIT-L and HIIT-S (MD = − 0.34, 95% CrI: −0.76 to 0.08). However, the HIIT-L versus MICT comparison was no longer statistically significant in a sensitivity analysis excluding Winding et al. (MD = − 0.35, 95% CrI: −0.74 to 0.05). For FPG, MICT (MD = − 0.77, 95% CrI: −1.10 to − 0.43), HIIT-S (MD = − 0.85, 95% CrI: −1.21 to − 0.52), and HIIT-L (MD = − 1.12, 95% CrI: −1.54 to − 0.70) all significantly reduced FPG levels relative to CON, whereas no significant differences were observed among the active exercise interventions. Based on the SUCRA rankings, HIIT-L ranked highest for both HbA1c (97.41%) and FPG (92.31%). For HOMA-IR, only HIIT-L showed a significant reduction compared with CON (MD = − 0.84, 95% CrI: −1.74 to − 0.06), whereas MICT and HIIT-S did not. However, because HOMA-IR was included post hoc as an exploratory outcome, and because the available evidence was limited and produced wide credible intervals, these findings should be interpreted cautiously.

**Conclusion:**

Current evidence indicates that MICT, HIIT-S, and HIIT-L are all associated with reductions in HbA1c and FPG compared with CON among individuals with T2D. HIIT-L had the highest ranking probabilities and the largest point estimates for both outcomes. Although the primary analysis suggested a greater reduction in HbA1c with HIIT-L than with MICT, this comparison was not robust to the exclusion of Winding et al. HIIT-S also improved glycemic control and may represent a practical alternative because each high-intensity work interval is shorter, although its feasibility and tolerability require direct evaluation. Future high-quality, volume-matched RCTs directly comparing HIIT-S and HIIT-L are needed to clarify the clinical utility of different aerobic HIIT protocols for T2D.

**Trial regisration:**

PROSPERO CRD420251175046.

**Supplementary Information:**

The online version contains supplementary material available at https://doi.org/10.1186/s13102-026-01921-4.

## Introduction

Type 2 diabetes (T2D) is one of the most prevalent chronic metabolic disorders globally, representing a profound public health challenge [1]. During the past 30 years, the global prevalence of diabetes mellitus (DM) has surged substantially, and DM ranks among the leading causes of death [2]. Currently, approximately one in eleven adults worldwide is affected by diabetes, with T2D accounting for roughly 90% of all cases [2]. Beyond its high prevalence rates, T2D is frequently linked to numerous complications. The majority of patients develop at least one DM-related complication, among which cardiovascular complications represent a primary cause of morbidity and mortality [2]. Effective glycemic control is a core objective in managing T2D. Glycated hemoglobin (HbA1c) and fasting plasma glucose (FPG) are among the most clinically relevant and frequently reported glycemic endpoints in individuals with T2D, reflecting long-term glycemic control and basal fasting glucose levels, respectively [3–5].

Furthermore, T2D is associated with substantial long-term clinical burden and complications, particularly when glycemic control is not sustained [6]. Therefore, identifying effective, feasible, and clinically useful non-pharmacological strategies is important for optimizing glycemic management among individuals with T2D [7, 8].

Exercise intervention is a crucial component of the comprehensive management of T2D, as it potentiates insulin sensitivity, augments glucose uptake, and optimizes glycemic control [9–11]. Currently, the most common exercise modalities for T2D primarily include moderate-intensity continuous training (MICT) and high-intensity interval training (HIIT) [9, 12]. Historically, MICT has been widely implemented in exercise management for DM. However, MICT generally requires longer continuous exercise sessions than low-volume HIIT protocols, which may represent a practical consideration for some patients [13]. In contrast, as a highly efficient, time-saving exercise protocol, HIIT has been increasingly explored recently. Prior evidence has demonstrated the positive effects of HIIT on improving HbA1c, FPG, and cardiorespiratory fitness [14]. Moreover, HIIT typically requires significantly less total exercise time than MICT while achieving similar or even superior metabolic benefits [10]. Certain studies have further suggested that HIIT may produce more favorable effects than MICT in improving glycemic control and maximal oxygen uptake among individuals with T2D [9, 15]. Beyond conventional glycemic outcomes, physical activity may also influence adipokines and inflammatory mediators that are involved in insulin resistance and the metabolic dysregulation of T2D [16].

However, HIIT is not a singular, homogeneous intervention. Various HIIT protocols exhibit marked differences in the duration of intervals, intensity of exercise, recovery mode, and total training volume [12, 15]. These variations may lead to distinct physiological stimuli and metabolic adaptations, thereby influencing the effects of diverse HIIT protocols on glycemic control [17]. Particularly within aerobic-based HIIT, protocols with varying interval durations may exhibit different clinical outcomes [18]. If all HIIT protocols are simply combined into one single intervention category, the true differences among different types of HIIT may be obscured. For instance, in a study involving individuals with T2D, Gentil et al. categorized aerobic HIIT into long-interval HIIT (HIIT-L) and short-interval HIIT (HIIT-S) based upon the duration of intervals. Their findings suggested that both modalities improve cardiorespiratory fitness. Nevertheless, HIIT-L produced more pronounced improvements in metabolic indicators such as HbA1c, suggesting its potential superiority over HIIT-S [19]. This finding further indicates that, even among various HIIT protocols, varying interval durations can elicit distinct metabolic adaptations. Therefore, further evidence is needed to refine this classification and enable direct comparisons between HIIT protocols.

Currently, multiple systematic reviews and meta-analyses have compared the effects of HIIT with MICT or non-exercising controls (CON) on indicators such as HbA1c, FPG, insulin resistance, and maximal oxygen uptake among individuals with T2D [11, 12, 20]. However, most studies have treated HIIT as a homogeneous entity, with relatively few further differentiating between its various modalities [10]. Evidence regarding the relative efficacy of diverse HIIT protocols, especially among different aerobic HIIT protocols, remains limited. Therefore, the optimal HIIT modality for improving glycemic control among individuals with T2D remains underexplored. Furthermore, traditional pairwise meta-analyses rely predominantly on direct comparative evidence. Consequently, it is infeasible to simultaneously integrate direct and indirect evidence across multiple interventions and systematically rank the relative efficacy of diverse interventions. By contrast, network meta-analysis (NMA) can integrate both direct and indirect evidence, compare multiple interventions simultaneously, and provide a ranking of interventions. Therefore, NMA is suitable for determining the relative benefits of various HIIT protocols.

Therefore, based on the duration of intervals, this research further stratified aerobic HIIT into HIIT-L and HIIT-S [18]. An NMA was subsequently conducted to compare the efficacy of various aerobic HIIT protocols, MICT, and CON on glycemic control among individuals with T2D.

## Methods

### Protocol and registration

This NMA was conducted in accordance with the Preferred Reporting Items for Systematic Reviews and Meta-Analyses extension statement for NMAs (PRISMA-NMA) guidelines [21]. Furthermore, it adhered to the methodological requirements outlined in the *Cochrane Handbook for Systematic Reviews of Interventions* [22] to ensure the transparency and scientific rigor of this research. The study protocol was prospectively registered in the International Prospective Register of Systematic Reviews (PROSPERO) (Registration number: CRD420251175046).

Several deviations from the registered protocol occurred during the conduct of this review. Although the PROSPERO record referred to both the original Cochrane risk-of-bias tool and RoB 2, only RoB 2 was used in the final review because all eligible studies were randomized trials and RoB 2 was considered the most appropriate current tool for this study design. In addition, although the registered protocol specified no language restrictions, the final review was restricted to English-language publications because translation resources were unavailable. Finally, HOMA-IR, which was not listed in the original registration, was added post hoc as an exploratory secondary outcome because it was commonly reported and was clinically relevant to insulin resistance. These deviations are reported transparently and are considered when interpreting the findings.

### Search strategy

PubMed, Embase, Cochrane Library, and Web of Science were searched from their inception to October 24, 2025, to comprehensively identify relevant randomized controlled trials (RCTs) fulfilling the inclusion criteria. Based upon the search characteristics of each database, the search strategies were developed using a combination of subject terms and free-text terms. The search terms primarily focused on type 2 diabetes mellitus and high-intensity interval training and were appropriately adapted to the indexing rules of each database. The reference lists of the included studies and relevant reviews were also manually screened to minimize the risk of missing pertinent studies. Detailed search strategies for each database are specified in Supplementary Table S1.

### Eligibility criteria

The inclusion and exclusion criteria for this study were formulated based upon the PICOS (Population, Intervention, Comparison, Outcome, and Study design) framework.

Inclusion criteria were outlined below: (i) Study population consisted of individuals aged ≥ eighteen years with a definitive clinical diagnosis of T2D, with no restrictions on sex, nationality, race, or disease duration; (ii) Regarding interventions, the intervention group received HIIT. This study only included training protocols exhibiting characteristics of aerobic HIIT (high-intensity, non-all-out interval training). The intensity of training was required to meet at least one of the following criteria: ≥90% VO₂max/VO₂peak, ≥ 90% HRmax/HRpeak, or 90%–120% of v/pVO₂max, MAP, or MAS. Training protocols involving all-out, Wingate, sprint interval training (SIT), or repeated sprint training (RST) were excluded. Furthermore, HIIT protocols were further classified based upon the duration of a single high-intensity interval. Protocols with work intervals of ≤ 60 s were classified as HIIT-S, whereas protocols with work intervals of ≥ 2 min were classified as HIIT-L. For progressive training designs, the categorization was based upon the interval duration of the primary training phase or the final stable phase [18, 19, 23]; (iii) Regarding comparison, the control interventions comprised usual care, non-exercise intervention, or MICT; (iv) Regarding outcomes, studies were required to report at least one eligible glycemic outcome. The prespecified primary outcomes were HbA1c and FPG. HOMA-IR was subsequently added post hoc as an exploratory secondary outcome because it was frequently reported in the eligible trials and was considered relevant to the assessment of insulin resistance. In this research, change values from pre-intervention to post-intervention were extracted for analysis to mitigate the impact of baseline intergroup imbalances on effect estimates. During the preliminary screening, 2-hour postprandial glucose was initially considered for inclusion. However, it was ultimately excluded from the final analysis owing to the limited number of eligible studies; (v) Eligible study designs were RCTs.

Exclusion criteria were outlined below: (i) studies including only individuals with prediabetes, or studies reporting aggregated data for T2D and prediabetes where independent data for T2D could not be extracted; (ii) studies using SIT as the primary intervention; (iii) reviews, systematic reviews, conference abstracts, case reports, animal experiments, and other non-original studies; (iv) duplicate publications; (v) studies with obvious data errors, unretrievable incomplete data, or a failure to report an eligible glycemic outcome; (vi) non-English publications, because translation resources were unavailable, and grey literature. The language restriction represented a deviation from the registered protocol.

### Study selection

Two investigators (Xuanming Hu and Meilin Wen) independently performed study selection and data extraction based upon pre-defined eligibility criteria. All retrieved records were imported into EndNote 21 for deduplication. A two-stage screening approach was subsequently adopted. First, titles and abstracts were screened to preliminarily exclude irrelevant studies. Second, complete texts of potentially eligible articles were downloaded and thoroughly reviewed to determine final inclusion. Any disagreements were resolved through discussions between the two investigators (Xuanming Hu and Meilin Wen) or by consulting a third researcher (Shunfang Liu).

### Data extraction

Data were extracted and recorded using a standardized Excel spreadsheet. The extracted information included first author, publication year, country, sample size, age of participants, duration of disease, intervention and control protocols, frequency and duration of training, and outcome measures. For continuous outcomes, the mean, standard deviation (SD), and sample size (N) for each group were extracted. Whenever available, change scores and their corresponding standard deviations (SDs) were extracted directly from the original studies. No additional imputation procedures were applied for outcome data. For multi-arm trials, all eligible intervention groups were included, and data were organized at the arm level. Multi-arm studies were modeled within the Bayesian network meta-analysis framework using the gemtc package, which appropriately accounts for the correlation between comparisons originating from the same study. This approach allowed for the simultaneous integration of all relevant comparisons in the NMA while appropriately accounting for the interrelations between diverse comparisons within the same study. Additionally, details regarding the duration of intervals and intensity of training were extracted to facilitate the further subclassification of aerobic HIIT into HIIT-L and HIIT-S. For studies including both prediabetes and type 2 diabetes, only data from participants with type 2 diabetes were extracted when reported separately. Detailed exercise-prescription characteristics, including work-interval duration, number of repetitions, recovery duration and mode, work-to-recovery ratio, total high-intensity time per session, total session duration, exercise frequency, intervention duration, and estimated weekly high-intensity training volume, were additionally extracted and are presented in Supplementary Table S2. Sabag et al. [24] and Way et al. [25] were identified as companion publications arising from the same randomized trial. They were therefore treated as a single study in the NMA, with the participant sample counted only once and each eligible outcome extracted from the publication providing the most complete relevant data. In Winding et al. [26], 29 unique participants were initially randomized. Following withdrawals and subsequent reallocation of six control participants to exercise groups, the original publication reported outcome data for 32 arm-period observations across CON, END, and HIIT. Because summary data restricted to the initially randomized parallel-group phase were unavailable, the arm-level outcome data reported by the authors were retained, while the study-level sample size was recorded as 29 unique randomized participants. The potential non-independence introduced by reallocation was examined through a sensitivity analysis excluding this trial.

### Study quality and risk of bias

The methodological quality of the included studies was assessed using the Cochrane risk-of-bias tool for RCTs (RoB 2). The assessment covered the following five domains: bias arising from the randomization process, bias owing to deviations from intended interventions, bias owing to missing outcome data, bias in measurements of the outcome, and bias in selection of the reported result. Each domain was judged as having a ‘low risk,’ ‘some concerns,’ or ‘high risk’ of bias. Subsequently, an overall risk-of-bias judgment was implemented for each study. No numerical summary score was calculated across the domains.

Two investigators (Xuanming Hu and Meilin Wen) independently assessed the risk of bias. Disagreements were resolved through discussion, with consultation of a third investigator (Shunfang Liu) when consensus could not be reached.

### Classification of interventions

In this study, following the classification approach of Stöggl et al. [18, 27], the interventions were first categorized into aerobic HIIT based upon the intensity of training. Subsequently, aerobic HIIT was further categorized into HIIT-L and HIIT-S based on the duration of each high-intensity interval. The specific classification criteria were outlined below:

First, high-intensity but non-all-out/maximal training protocols were classified as aerobic HIIT [18, 27, 28]. Specifically, the included aerobic HIIT protocols were required to meet at least one of the following intensity criteria: ≥90% VO2max, ≥ 90% HRmax/HRpeak, or 90%–120% of v/pVO2max, MAP, or MAS. Protocols clearly identified as all-out, Wingate, SIT, or RST were not classified as aerobic HIIT [18, 28, 29].

Secondly, aerobic HIIT was further classified into HIIT-S and HIIT-L based on the durations of each high-intensity interval. Among these, HIIT-S was defined as training protocols with a single high-intensity interval of ≤ 60 s, with typical formats including 20 s, 30 s, and 1 min intervals [19, 30–32]. HIIT-L was defined as training protocols with a single high-intensity interval of ≥ 2 min, with typical formats including 2 min and 4 min intervals [19, 23, 32, 33].

This classification approach largely aligns with the distinction of aerobic HIIT in prior evidence [18], in which short aerobic intervals typically ranged from 15 to 60 s, whereas long aerobic intervals typically ranged from 2 to 10 min. The selected thresholds were intended to distinguish two commonly recognized aerobic HIIT structures rather than to imply that interval duration alone determines the physiological stimulus. Short intervals of ≤ 60 s generally involve repeated brief exposures to high intensity, whereas intervals of ≥ 2 min permit a more prolonged cardiovascular and metabolic response during each work bout. However, the physiological load generated by either format also depends on recovery duration and intensity, work-to-recovery ratio, number of repetitions, accumulated high-intensity time, total session duration, and weekly training volume. Therefore, the HIIT-S and HIIT-L categories should be interpreted as pragmatic protocol classifications rather than physiologically homogeneous interventions. Notably, the 1 min interval represents the upper boundary of short intervals. However, previous studies have treated 1-min protocols as HIIT-S. Therefore, this study also categorized them into HIIT-S (e.g., the 10 × 1 min HIIT protocol) [34]. Protocols with work intervals between 60 and 119 s were not assigned to either HIIT-S or HIIT-L in the primary analysis, because they did not meet the predefined interval-duration thresholds. The detailed classification criteria are presented in Table 1.

**Table 1:** Classification criteria for aerobic HIIT protocols

Classification Dimension	HIIT-S	HIIT-L
Prerequisites	aerobic HIIT; high-intensity, non-all-out/maximal	aerobic HIIT; high-intensity, non-all-out/maximal
Intensity criteria	≥ 90% VO_2_max; ≥ 90% HRmax/HRpeak; or 90%–120% of v/pVO_2_max, MAP, or MAS	≥ 90% VO_2_max; ≥ 90% HRmax/ HRpeak; or 90%–120% of v/pVO_2_max, MAP, or MAS
Interval duration	≤ 60 s	≥ 2 min
Typical protocols	Twenty seconds, thirty seconds, one minute	Two minutes, four minutes
Remarks	1-min intervals are classified under HIIT-S	Consistent with 2–10 min long aerobic intervals

Protocols involving all-out, Wingate, sprint interval training (SIT), or repeated sprint training (RST) are not included in the category of aerobic HIIT. Protocols with work intervals between 60 and 119 s were treated as borderline protocols and were excluded from the primary classification-based analysis

### Statistical analysis

A Bayesian NMA was implemented using the gemtc package in R version 4.5.2 [35]. The Bayesian models were fitted using four Markov chains with 20,000 iterations each. Default non-informative prior distributions implemented in the gemtc package were used for all model parameters. Network plots were generated to illustrate the available comparisons among the interventions. Model fit was evaluated using the deviance information criterion (DIC) and the ratio of residual deviance to the number of data points. The convergence of models was assessed through trace plots and the Gelman–Rubin diagnostic. When closed loops were present in the network, the node-splitting method was applied to examine local inconsistency.

The relative efficacy of interventions was compared using league tables, with effect sizes estimated as mean differences (MDs) and their corresponding 95% credible intervals (CrIs). The surface under the cumulative ranking curve (SUCRA) was calculated to rank the relative effectiveness of the interventions. Higher SUCRA values indicated a greater probability of ranking among the more effective interventions. The plausibility of the transitivity assumption was assessed by comparing potential effect modifiers across treatment comparisons, including participant age, baseline glycemic status, disease duration, medication use, intervention duration, exercise frequency, recovery structure, and total training volume. Participant characteristics were broadly comparable; however, exercise-prescription variables differed across trials, and residual intransitivity related to exercise dose could not be excluded. Furthermore, sensitivity analyses were conducted using fixed-effect models, and the results were compared with those from the random-effects models to assess the robustness of the findings. An additional sensitivity analysis excluding Liu et al. was conducted because this study differed from the remaining studies in intervention duration (3 weeks) and enrolled both participants with prediabetes and type 2 diabetes. An additional sensitivity analysis excluding Winding et al. was conducted to evaluate the influence of the non-independent arm-period observations arising from participant reallocation. Reporting bias and small-study effects were not formally assessed because the number of studies available within each outcome network and direct comparison was limited.

## Results

### Study selection

In total, 2,999 records were initially identified from four English databases: Web of Science (*n* = 648), PubMed (*n* = 532), Cochrane Library (*n* = 628), and Embase (*n* = 1,191). After 1217 duplicate records were removed using EndNote 21, the remaining 1,782 records were screened by reviewing their titles and abstracts. Subsequently, 1722 records were excluded. Complete texts were obtained for 54 of the remaining 60 reports, while 6 were excluded owing to unavailable complete texts. Finally, complete texts of 54 articles were assessed for eligibility.

**Fig. 1:**
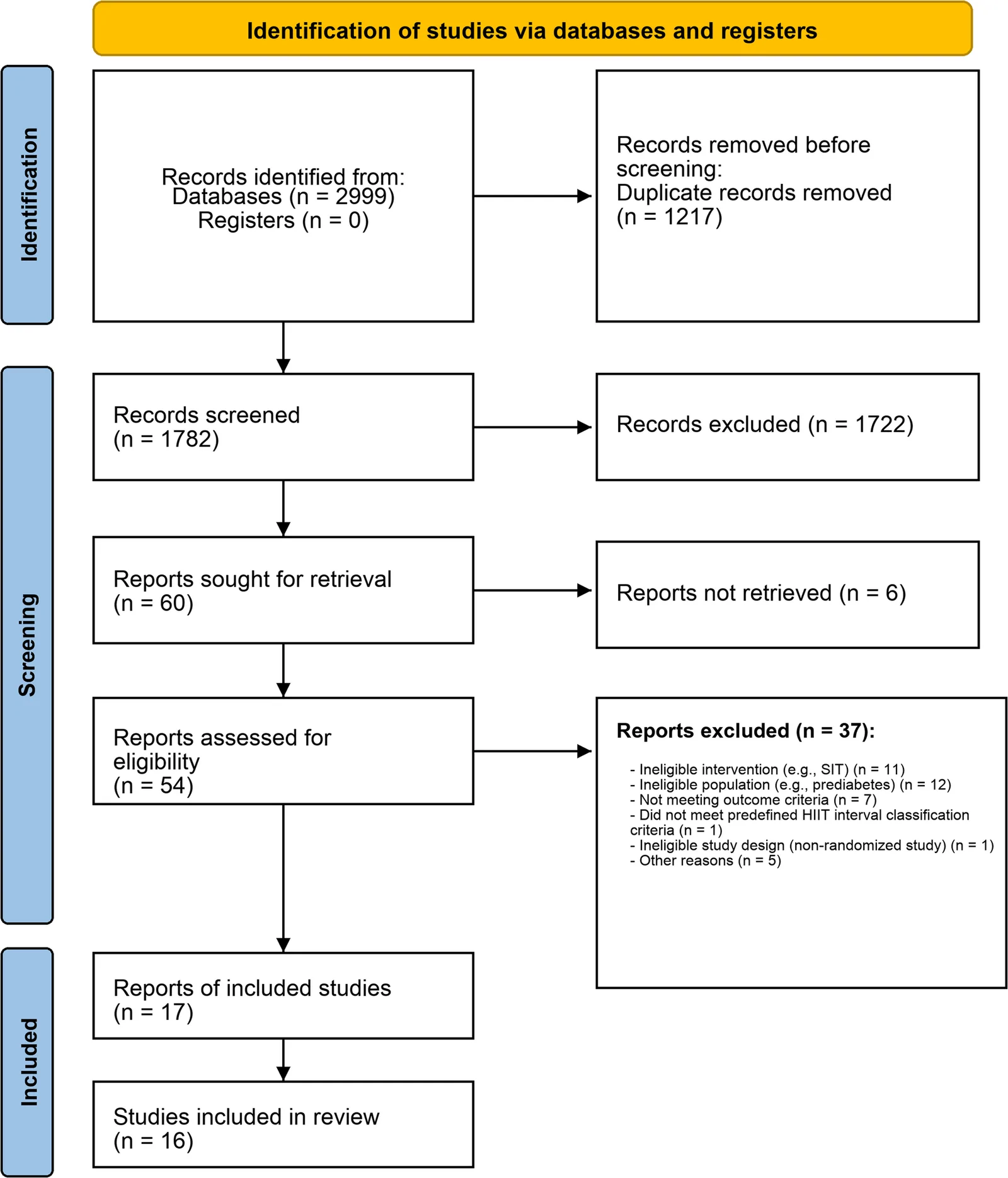
Flowchart of literature screening

Based on the eligibility criteria, 37 full-text articles were excluded for the following reasons: ineligible interventions (*n* = 11), ineligible populations (*n* = 12), failure to meet outcome criteria (*n* = 7), failure to meet the predefined HIIT interval-classification criteria (*n* = 1), non-randomized allocation (*n* = 1), and other reasons (*n* = 5). Ultimately, 16 unique trials reported across 17 publications [13, 19, 24–26, 36–47], involving 744 participants with T2D, were included. The study-selection process is shown in Fig. 1.

### Features of included studies

Sixteen unique trials reported across 17 publications [13, 19, 24–26, 36–47] were included. These studies were published between 2014 and 2025, and all participants were adults with T2D. Based on the predefined criteria, HIIT interventions were classified as HIIT-S or HIIT-L according to work-interval duration and exercise intensity; the remaining interventions were classified as MICT or CON. Intervention duration ranged from 3 to 12 weeks. Of the 16 trials, 15 reported HbA1c, 13 reported FPG, and 7 reported HOMA-IR. Study characteristics are presented in Table 2. Exercise prescriptions varied substantially in the number of repetitions, recovery structure, accumulated high-intensity time per session, total session duration, training frequency, and intervention duration. Accumulated high-intensity work ranged from approximately 4 to 17 min per session for HIIT-S and from 4 to 16 min per session for HIIT-L. Energy expenditure and time spent above specific physiological thresholds were not reported consistently and could not be compared quantitatively. A descriptive summary is provided in Supplementary Table S2.

**Table 2:** Basic features of included studies

No.	Study	Country	Total (*n*)	Arms included	Classification	HIIT Protocol	Duration	Outcomes
1	Winding 2018 [26]	Denmark	29	CON, MICT, HIIT-S	HIIT-S	10 × 1 min at 95% Wpeak with 1 min active recovery at 20% Wpeak, 20 min/session, 3 sessions/week	Eleven weeks	HbA1c, FPG, HOMA-IR
2	Li 2022 [36]	China	39	CON, MICT, HIIT-S	HIIT-S	5-min warm-up, followed by 15 min HIIT including 8 min at 80%–95% intensity and 7 min active recovery at ~ 25% intensity, plus 5-min finishing activities; 5 sessions/week	Twelve weeks	HbA1c, FPG
3	Cassidy 2019 [37]	United Kingdom	22	CON, HIIT-L	HIIT-L	5 progressive intervals/session at RPE 16–17, from 2 min in week 1 to 3 min 50 s in week 12, with 3-min recovery between intervals; 3 sessions/week	Twelve weeks	HbA1c
4	Mitranun 2014 [38]	Thailand	43	CON, MICT, HIIT-S	HIIT-S	4–6 × 1 min at 80%–85% VO₂peak with 4 min active recovery at 50%–60% VO₂peak; 30 min/session; 3 sessions/week	Twelve weeks	HbA1c, FPG, HOMA-IR
5	Ahmad 2023 [39]	Egypt	72	CON, HIIT-L (low-volume), HIIT-L (high-volume)	HIIT-L	Low-volume: 2 × 4 min at 85%–90% peak HR with 3-min active recovery at 65%–75% peak HR; High-volume: 4 × 4 min at 85%–90% peak HR with 3-min active recovery at 65%–75% peak HR; 3 sessions/week	Twelve weeks	HbA1c, FPG
6	Alizadeh 2025 [40]	Iran	18	MICT, HIIT-S	HIIT-S	7 × 1 min at 90%–95% HRR, with 2 min active recovery at 60%–70% HRR; 3 sessions/week	Eight weeks	HbA1c, FPG
7	Sabouri 2021 [41]	Iran	29	CON, HIIT-S	HIIT-S	10 × 1 min at 85%–90% HRmax with 1 min active recovery; 5-min warm-up and 5-min cool-down at ~ 40% HRmax; 3 sessions/week	Twelve weeks	HbA1c, FPG, HOMA-IR
8	Sabag/Way 2020 [24, 25]	Australia	35	CON, MICT, HIIT-L	HIIT-L	1 × 4 min at 90% VO₂peak; 10-min warm-up and 5-min cool-down at 50% VO₂peak; 3 sessions/week	Twelve weeks	HbA1c, FPG
9	Mohammadi zadeh 2022 [42]	Iran	34	MICT, HIIT-S	HIIT-S	8–12 × 1 min at 90%–100% HRmax with 2 min active recovery at 60%–70% HRmax; 4–5-min warm-up and 4-min cool-down; 4 sessions/week	Twelve weeks	HbA1c
10	Kazemi 2024 [43]	Iran	33	CON, HIIT-S	HIIT-S	10 × 1 min at 90% HRmax with 1 min active recovery at 50 W; 3-min warm-up and 3-min cool-down at 50 W; 3 sessions/week	Twelve weeks	HbA1c, FPG
11	Gentil 2023 [19]	Brazil	44	MICT, HIIT-L, HIIT-S	HIIT-L, HIIT-S	HIIT-L: 5 × 2 min at 100% vVO₂max with 2 min passive recovery; 2-min warm-up and 2-min cool-down at 50% vVO₂max; HIIT-S: 20 × 30 s at 100% vVO₂max with 30 s passive recovery; 2-min warm-up and 2-min cool-down at 50% vVO₂max ; 2 sessions/week	Eight weeks	HbA1c, FPG
12	Niyazi 2024 [13]	Iran	36	CON, MICT, HIIT-L	HIIT-L	4 × 4 min at 85%–95% HRmax with 3 min active recovery at 50%–60% HRmax; 10-min warm-up and 5-min cool-down; 3 sessions/week	Twelve weeks	HbA1c, FPG, HOMA-IR
13	Findikoglu 2023 [44]	Turkey	60	CON, MICT, HIIT-S	HIIT-S	8–16 × 1 min at 90% VO₂peak with 120 s low-intensity recovery at 30% VO₂peak; 5-min warm-up and 5-min cool-down at 20 W; 3 sessions/week	Twelve weeks	HbA1c, FPG, HOMA-IR
14	Riahy 2024 [45]	Iran	37	CON, MICT, HIIT-L	HIIT-L	4 × 4 min at 85%–95% HRmax with 3 min active recovery at 50%–60% HRmax; 10-min standardized warm-up at 50%–60% HRmax and 5-min cool-down; 3 sessions/week	Twelve weeks	HbA1c, HOMA-IR
15	Abdi 2021 [46]	Iran	30	CON, HIIT-L	HIIT-L	4 × 4 min at 85%–95% HRmax with 3 min active recovery at 50%–60% HRmax; 10-min warm-up and 5-min cool-down at 40% HRmax; 3 sessions/week	Twelve weeks	HbA1c, FPG, HOMA-IR
16	Liu 2020 [47]	China	183	MICT, HIIT-S	HIIT-S	20 × 50 s at 90% HRmax with 10 s passive recovery at 20% HRmax on a cycle ergometer; ≥3 sessions/week	Three weeks	FPG

Winding et al. initially randomized 29 participants; after six control participants were reallocated to exercise groups, the published outcome data comprised 32 arm-level observations (CON = 7, END = 12, HIIT = 13)

*CON* control group, *MICT* moderate-intensity continuous training, *HIIT-S* short-interval HIIT, *HIIT-L* long-interval HIIT, *HbA1c* glycated hemoglobin, *FPG* fasting plasma glucose, *HOMA-IR* homeostatic model assessment of insulin resistance

### Risk of bias

**Fig. 2:**
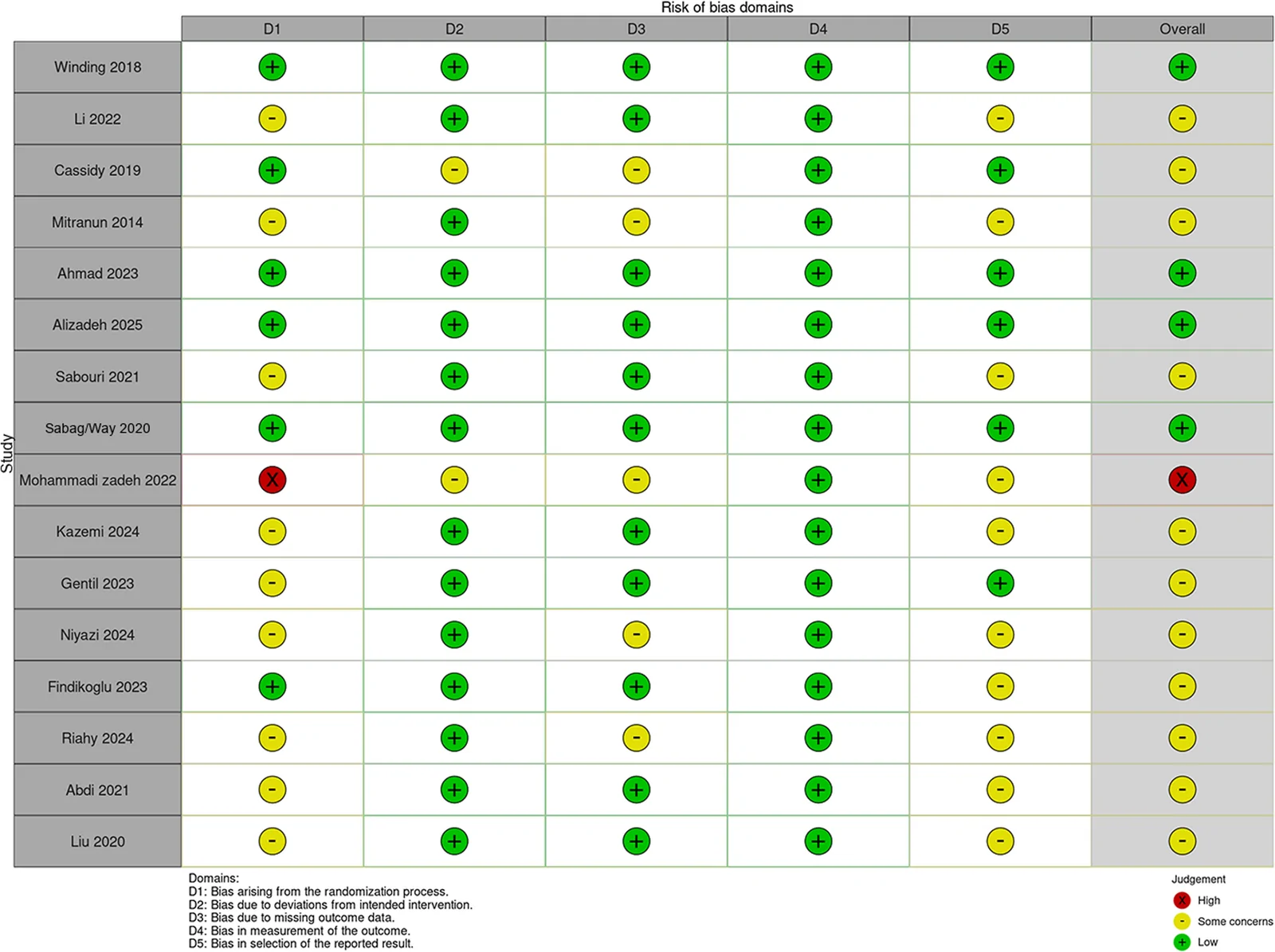
Quality assessment of included studies

The risk of bias for the included studies was assessed using the Cochrane RoB 2 tool, with the results presented in Fig. 2. Overall, the majority of the included studies were rated as having a low risk of bias or some concerns, while one trial was judged to have an overall high risk of bias [42]. The study judged as having an overall high risk of bias was primarily rated as high risk in the randomization process because reporting of random-sequence generation and allocation concealment was insufficient. It was also rated as having some concerns regarding deviations from intended interventions, handling of missing outcome data, and selective reporting of results [42]. Collectively, the primary sources of bias across the studies were concentrated in the domains of the randomization process, missing outcome data, and selective reporting of results. By contrast, given that outcomes like HbA1c, FPG, and HOMA-IR were mostly objective biochemical indicators, the overall risk of bias in the domain of measurement of the outcome was generally low.

### Results of NMA

#### HbA1c

Fifteen studies reported HbA1c and were included in this outcome network, involving four interventions: CON, MICT, HIIT-S, and HIIT-L. The random-effects model showed acceptable model fit, with a residual deviance ratio of 0.85 and a DIC of 59.12. The estimated between-study heterogeneity was τ = 0.30 (95% CrI: 0.16 to 0.49). Model convergence was satisfactory according to the Gelman–Rubin diagnostic.

Compared with CON, all three exercise interventions were associated with significant reductions in HbA1c: MICT (MD = − 0.47, 95% CrI: −0.77 to − 0.17), HIIT-S (MD = − 0.50, 95% CrI: −0.84 to − 0.18), and HIIT-L (MD = − 0.85, 95% CrI: −1.16 to − 0.55). HIIT-L also showed a greater reduction in HbA1c than MICT (MD = − 0.38, 95% CrI: −0.76 to − 0.01). However, no significant difference was observed between HIIT-L and HIIT-S (MD = − 0.34, 95% CrI: −0.76 to 0.08) or between HIIT-S and MICT (MD = − 0.04, 95% CrI: −0.36 to 0.28).

**Fig. 3:**
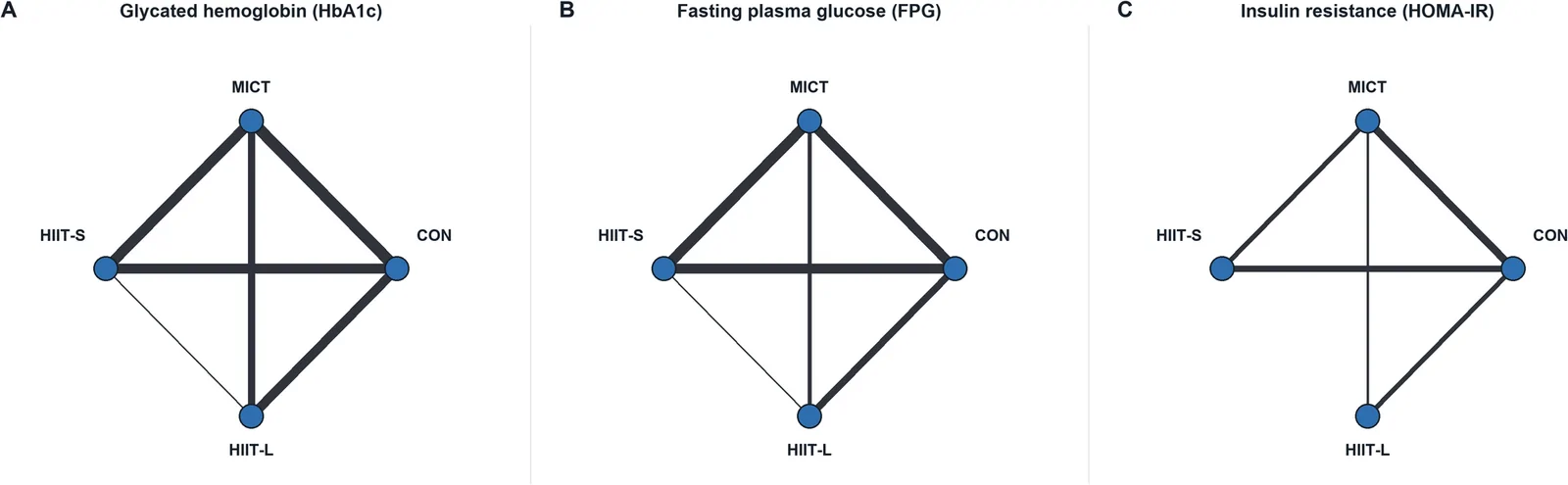
Network plots for different outcomes Note: **A** = HbA1c; **B** = FPG; **C** = HOMA-IR

Based on SUCRA values, HIIT-L ranked highest (97.41%), followed by HIIT-S (54.75%), MICT (47.47%), and CON (0.12%). No significant local inconsistency was detected in the node-splitting analysis, with all p values greater than 0.05. The corresponding network plot, league table, model fit and convergence diagnostics, node-splitting analysis, and SUCRA ranking plot are presented in Fig. 3; Table 3, Supplementary Table S3, Supplementary Figure S1, and Supplementary Figure S2.

**Table 3:** League table of HbA1c

CON	MICT	HIIT-S	HIIT-L
CON	−0.47 (− 0.77, − 0.17)	−0.50 (− 0.84, − 0.18)	−0.85 (− 1.16, − 0.55)
0.47 (0.17, 0.77)	MICT	−0.04 (− 0.36, 0.28)	−0.38 (− 0.76, − 0.01)
0.50 (0.18, 0.84)	0.04 (− 0.28, 0.36)	HIIT-S	−0.34 (− 0.76, 0.08)
0.85 (0.55, 1.16)	0.38 (0.01, 0.76)	0.34 (− 0.08, 0.76)	HIIT-L

*CON *non-exercise control, *MICT* moderate-intensity continuous training, *HIIT-S* short-interval high-intensity interval training, *HIIT-L* long-interval HIIT

#### FPG

Thirteen unique trials, reported across 14 publications [13, 19, 24–26, 36, 38–41, 43, 44, 46, 47], were included in the FPG network and involved four interventions: CON, MICT, HIIT-S, and HIIT-L. The random-effects model showed acceptable model fit for FPG, with a residual deviance ratio of 0.82 and a DIC of 44.77. The model-based estimate suggested limited between-study heterogeneity (τ = 0.15, 95% CrI: 0.01–0.48; I² = 0%). Meanwhile, the model demonstrated satisfactory convergence (PSRF = 1.00). The NMA showed that MICT (MD = − 0.77, 95% CrI: −1.10 to − 0.43), HIIT-S (MD = − 0.85, 95% CrI: −1.21 to − 0.52), and HIIT-L (MD = − 1.12, 95% CrI: −1.54 to − 0.70) all significantly reduced FPG levels relative to CON. Among these interventions, HIIT-L showed the largest estimated reduction. Nevertheless, no significant differences were detected in the pairwise comparisons among MICT, HIIT-S, and HIIT-L.

Based on the results of SUCRA, HIIT-L ranked the highest (92.31%), followed by HIIT-S (62.34%) and MICT (45.33%). Meanwhile, CON ranked the lowest (0.02%). No significant local inconsistency was identified by node-splitting analysis, suggesting that the network evidence for the FPG outcome was relatively stable. The corresponding network plot, league table, model fit and convergence diagnostics, node-splitting analysis, and SUCRA ranking plot are presented in Fig. 3; Table 4, Supplementary Table S3, Supplementary Figure S1, and Supplementary Figure S2.

**Table 4:** League table of FPG

CON	MICT	HIIT-S	HIIT-L
CON	-0.77 (-1.1, -0.43)	-0.85 (-1.21, -0.52)	-1.12 (-1.54, -0.7)
0.77 (0.43, 1.1)	MICT	-0.08 (-0.4, 0.23)	-0.36 (-0.85, 0.15)
0.85 (0.52, 1.21)	0.08 (-0.23, 0.4)	HIIT-S	-0.27 (-0.78, 0.26)
1.12 (0.7, 1.54)	0.36 (-0.15, 0.85)	0.27 (-0.26, 0.78)	HIIT-L

*CON* non-exercise control, *MICT* moderate-intensity continuous training, *HIIT-S* short-interval high-intensity interval training, *HIIT-L* long-interval HIIT

#### HOMA-IR

Seven studies reported HOMA-IR [13, 26, 38, 41, 44–46], involving four interventions: CON, MICT, HIIT-S, and HIIT-L. The random-effects model showed acceptable model fit for HOMA-IR, with a residual deviance ratio of 0.88 and a DIC of 29.92. The model-based estimate suggested moderate uncertainty in between-study heterogeneity (τ = 0.48, 95% CrI: 0.03–1.22; I² = 0%). Meanwhile, the model demonstrated satisfactory convergence (PSRF = 1.00).

The NMA showed that HIIT-L significantly reduced HOMA-IR relative to CON (MD = − 0.84, 95% CrI: −1.74 to − 0.06). However, the effects of MICT (MD = − 0.42, 95% CrI: −1.09 to 0.38) and HIIT-S (MD = − 0.71, 95% CrI: −1.61 to 0.25) were not statistically significant relative to CON. No significant differences were observed among the three active exercise interventions.

Based on the results of SUCRA, HIIT-L ranked the highest (82.34%), followed by HIIT-S (69.43%) and MICT (41.36%). Meanwhile, CON ranked the lowest (6.88%). Node-splitting analysis did not identify statistically significant local inconsistency within the HOMA-IR network. However, the comparison between MICT and HIIT-L showed a borderline inconsistency signal (*P* = 0.066), suggesting that these findings should be interpreted cautiously. Therefore, the HOMA-IR findings should be interpreted cautiously because of the limited number of studies, wide credible intervals, the borderline inconsistency signal, and uncertainty in the network estimates. The corresponding network plot, league table, model fit and convergence diagnostics, node-splitting analysis, and SUCRA ranking plot are presented in Fig. 3; Table 5, Supplementary Table S3, Supplementary Figure S1, and Supplementary Figure S2.

**Table 5:** League table of HOMA-IR

CON	MICT	HIIT-S	HIIT-L
CON	-0.42 (-1.09, 0.38)	-0.71 (-1.61, 0.25)	-0.84 (-1.74, -0.06)
0.42 (-0.38, 1.09)	MICT	-0.3 (-1.29, 0.65)	-0.42 (-1.45, 0.34)
0.71 (-0.25, 1.61)	0.3 (-0.65, 1.29)	HIIT-S	-0.14 (-1.4, 0.95)
0.84 (0.06, 1.74)	0.42 (-0.34, 1.45)	0.14 (-0.95, 1.4)	HIIT-L

*CON* non-exercise control, *MICT* moderate-intensity continuous training, *HIIT-S* short-interval high-intensity interval training, *HIIT-L* long-interval HIIT

### Sensitivity analysis

To assess the impact of model selection on the stability of these results, sensitivity analyses were performed using fixed-effect models. Detailed results of the fixed-effect model sensitivity analysis are presented in Supplementary Table S4. For HbA1c, the fixed-effect model showed broadly consistent findings with the random-effects model. Compared with CON, MICT (MD = − 0.41, 95% CrI: −0.59 to − 0.23), HIIT-S (MD = − 0.46, 95% CrI: −0.65 to − 0.26), and HIIT-L (MD = − 0.78, 95% CrI: −0.93 to − 0.64) all significantly reduced HbA1c levels. HIIT-L remained the highest-ranked intervention. The fixed-effect model suggested significant differences between HIIT-L and MICT (MD = − 0.37, 95% CrI: −0.59 to − 0.16) and between HIIT-L and HIIT-S (MD = − 0.33, 95% CrI: −0.56 to − 0.09), whereas the HIIT-L versus HIIT-S comparison remained non-significant in the random-effects model. Therefore, the comparative advantage of HIIT-L over HIIT-S should be interpreted cautiously.

For FPG, the results from the fixed-effect model were highly consistent with those from the random-effects model. Relative to CON, MICT (MD = − 0.77, 95% CrI: −1.06 to − 0.48), HIIT-S (MD = − 0.85, 95% CrI: −1.14 to − 0.57), and HIIT-L (MD = − 1.13, 95% CrI: −1.47 to − 0.78) all significantly reduced FPG levels. No significant differences were observed among the active exercise interventions, and the ranking of the interventions remained unchanged, with HIIT-L ranked highest, followed by HIIT-S, MICT, and CON. For HbA1c, a local inconsistency signal was observed for the MICT versus HIIT-L comparison under the fixed-effect model, whereas no statistically significant local inconsistency was identified under the primary random-effects model.

For HOMA-IR, the fixed-effect model suggested significant reductions for all three exercise interventions relative to CON. However, this differed from the random-effects model, in which only HIIT-L showed a significant reduction relative to CON. In addition, significant local inconsistency was detected for the MICT versus HIIT-L comparison in the fixed-effect model. Therefore, the HOMA-IR findings were considered sensitive to model specification and network consistency and should be interpreted cautiously as exploratory findings. Overall, the fixed-effect sensitivity analyses supported the robustness of the main conclusions for HbA1c and FPG, while reinforcing the uncertainty of the HOMA-IR results. Given the potential clinical heterogeneity among included studies, random-effects models were retained as the primary analytical models.

Because Liu et al. differed from the remaining studies in both intervention duration (3 weeks) and participant characteristics, an additional sensitivity analysis excluding this study was conducted. After exclusion of Liu et al. [47], the pooled effect estimates, treatment rankings, and overall conclusions remained materially unchanged across all outcomes, indicating that the primary findings were robust and were not driven by this study. An additional sensitivity analysis excluding Winding et al. was conducted to evaluate the potential influence of its non-independent arm-period observations. For FPG and HOMA-IR, the effect estimates, statistical significance, and treatment rankings remained materially unchanged after exclusion of this trial. For HbA1c, all three exercise interventions remained significantly more effective than CON, and HIIT-L retained the highest ranking. However, the comparison between HIIT-L and MICT was attenuated and was no longer statistically significant after exclusion of Winding et al. (MD = − 0.35, 95% CrI: −0.74 to 0.05). These findings indicate that the overall conclusions for FPG and HOMA-IR were robust, whereas the evidence supporting the superiority of HIIT-L over MICT for HbA1c was sensitive to the inclusion of Winding et al. (Supplementary Table S5).

### Summary of findings

Overall, MICT, HIIT-S, and HIIT-L were all associated with reductions in HbA1c and FPG relative to CON among individuals with T2D. Among the active interventions, HIIT-L showed the largest estimated reductions and the highest SUCRA rankings for both primary outcomes. In the primary analysis, HIIT-L was significantly more effective than MICT in reducing HbA1c; however, this comparison was no longer statistically significant after exclusion of Winding et al., indicating that this specific finding was sensitive to the inclusion of that trial. In addition, HIIT-L was not significantly superior to HIIT-S in the random-effects model, and no significant differences were observed among the active interventions for FPG. For HOMA-IR, HIIT-L showed a significant reduction compared with CON, but the evidence was less robust because of sparse data, wide credible intervals, and sensitivity to model specification. Therefore, the main findings should be based primarily on HbA1c and FPG, while HOMA-IR should be interpreted as an exploratory outcome.

## Discussion

In this study, a Bayesian NMA was conducted to systematically compare the efficacy of MICT, HIIT-S, and HIIT-L on glycemic outcomes among individuals with T2D. The results showed that MICT, HIIT-S, and HIIT-L were all associated with reductions in the levels of HbA1c and FPG relative to conventional control. Notably, HIIT-L achieved the highest SUCRA ranking and generally showed the largest effect estimates for both HbA1c and FPG. Conversely, for HOMA-IR, although HIIT-L showed the highest ranking probability, the differences among active interventions were not statistically significant. Moreover, no statistically significant local inconsistency was detected by node-splitting analysis (all *P* > 0.05), although the comparison between MICT and HIIT-L showed a borderline inconsistency signal (*P* = 0.066). Together with the limited number of studies and wide credible intervals, these findings indicate considerable uncertainty and should therefore be interpreted cautiously. Overall, all three exercise interventions were associated with improvements in HbA1c and FPG relative to CON. HIIT-L produced the most favorable ranking and the largest point estimates. In the primary analysis, its comparative advantage was statistically supported only for HbA1c versus MICT; however, this comparison was no longer statistically significant after exclusion of Winding et al. Therefore, the apparent superiority of HIIT-L over MICT for HbA1c should be interpreted cautiously. The estimated HbA1c reductions relative to CON were approximately 0.5–0.9% points and may be clinically meaningful for many individuals with T2D. However, their clinical importance is likely to vary according to baseline HbA1c, medication use, disease duration, and individual treatment goals.

HIIT-L produced the largest point estimates and the highest ranking probabilities for HbA1c and FPG. However, these findings should not be attributed solely to the duration of individual high-intensity intervals. The included interventions were generally not matched for total external work, energy expenditure, accumulated high-intensity time, or total weekly training volume. The protocols also differed in the number of repetitions, recovery duration and intensity, work-to-recovery ratio, total session duration, weekly training frequency, and intervention duration. Consequently, some HIIT-L protocols may have provided a greater accumulated high-intensity stimulus, whereas MICT protocols generally involved longer durations of lower-intensity exercise. These differences may influence cumulative muscle contraction, glycogen utilization, post-exercise glucose uptake, and total energy expenditure. Therefore, the observed estimates may partly reflect unequal physiological and training doses rather than an independent effect of interval duration. Detailed protocol-level differences are summarized in Supplementary Table S2, and the apparent advantage of HIIT-L should consequently be interpreted as hypothesis-generating.

The present NMA did not directly assess physiological or molecular mechanisms. However, previous experimental studies have suggested that exercise training may influence skeletal-muscle glucose uptake, glycogen utilization, mitochondrial function, and insulin sensitivity [48–53]. Clinical studies and evidence syntheses have also reported improvements in glycemic and related metabolic outcomes following HIIT and other structured exercise interventions among individuals with T2D [14, 54, 55]. These mechanisms provide possible biological explanations for exercise-related improvements in glycemic control, but they were not directly evaluated in the included trials. Accordingly, the present NMA cannot determine whether the observed effects were mediated by changes in glucose uptake, glycogen utilization, mitochondrial adaptations, or insulin sensitivity. No interval-duration-specific mechanism can therefore be inferred from the current evidence, particularly because interval duration was accompanied by differences in accumulated high-intensity time, recovery structure, and weekly exercise volume.

Related evidence outside the eligibility criteria of this NMA provides broader biological context for exercise-related metabolic adaptations. A literature review summarized the potential effects of physical activity on adipokines in individuals with T2D [16]. Other studies reported changes in retinol-binding protein 4 and tumor necrosis factor-α after circuit resistance training combined with Zataria multiflora supplementation in postmenopausal women [56], discussed the possible role of Meteorin-like protein in exercise-related adaptations in T2D [57], examined Gremlin-1 and macrophage migration inhibitory factor after resistance training in men with obesity [58], reported reductions in WISP-1 and WISP-2 after circuit resistance training in individuals with T2D [59], and proposed fetuin-B as a potential link between insulin resistance and exercise [60]. Because these publications involved different populations, exercise modalities, and, in one study, a nutritional co-intervention, they do not demonstrate mechanisms specific to HIIT interval duration.

No clear differences were observed among the three active exercise interventions for HOMA-IR. HOMA-IR is a surrogate index derived from fasting glucose and insulin and may be influenced by baseline insulin concentrations, medication use, testing conditions, and interindividual variability. Moreover, previous meta-analyses indicate that exercise-related changes in insulin sensitivity may vary according to the population and exercise modality examined [61, 62]. Therefore, the present HOMA-IR findings should not be regarded as conclusive evidence of a differential treatment effect and should instead be interpreted as exploratory.

Taken together, the apparent advantage of HIIT-L should be regarded as hypothesis-generating rather than mechanistically established. Volume-matched, head-to-head trials directly comparing HIIT-L and HIIT-S are required to determine whether interval duration itself has an independent effect on glycemic control [12, 18, 23]. Meanwhile, HIIT-S was also associated with significant reductions in HbA1c and FPG relative to CON. Although the point estimates and ranking probabilities favored HIIT-L, the comparative effectiveness of HIIT-S and HIIT-L remained uncertain under the primary random-effects model. By dividing total high-intensity exercise time into shorter intervals of 30–60 s, HIIT-S reduces the duration of continuous high-intensity exposure during each individual bout [19, 30, 32, 34]. Previous meta-analyses have also reported favorable body-composition and cardiorespiratory-fitness responses to interval training compared with MICT in broader adult populations [63, 64]. Nevertheless, whether the shorter work intervals of HIIT-S translate into better adherence, tolerability, or patient acceptability among individuals with T2D requires direct evaluation.

Finally, MICT remained effective in reducing HbA1c and FPG and continues to represent an established component of exercise management for individuals with T2D. Its clinical implementation is supported by established exercise recommendations and its adaptability to a wide range of patient characteristics and clinical settings [65]. Although adherence and safety were not consistently reported across all included studies, the majority of trials described satisfactory intervention adherence and no serious exercise-related adverse events. When adverse events were reported, they were generally mild and transient (e.g., temporary fatigue or musculoskeletal discomfort), suggesting that supervised HIIT-S and HIIT-L were generally feasible in the populations studied. Nevertheless, because safety and adherence outcomes were not uniformly reported, more standardized reporting is needed in future trials. From a clinical perspective, the present ranking results should not be interpreted as supporting the universal prescription of HIIT-L for all individuals with T2D. Suitability for longer high-intensity intervals is likely to depend on age, baseline cardiorespiratory fitness, disease duration, glycemic stability, medication use, cardiovascular risk, musculoskeletal limitations, peripheral neuropathy, and previous exercise experience. HIIT-L may be most appropriate for medically stable individuals who have adequate functional capacity, previous exercise exposure, and access to professional supervision. In contrast, HIIT-S may represent a more acceptable starting option for individuals with lower fitness, limited tolerance of sustained high-intensity effort, or concerns regarding fatigue and perceived exertion, because the continuous duration of each high-intensity bout is shorter. Nevertheless, shorter intervals are not necessarily lower in total physiological load, and their safety and tolerability remain dependent on intensity, recovery structure, and accumulated work. Clinical implementation should therefore involve individualized screening, gradual progression from moderate-intensity exercise or shorter intervals, monitoring of symptoms and glycemic responses, and appropriate adjustment of exercise intensity and recovery duration. Supervised initiation may be particularly important for individuals with cardiovascular complications, autonomic dysfunction, neuropathy, poor glycemic stability, or limited prior exposure to vigorous exercise. The present NMA supports HIIT-L as a potentially effective option rather than a universally preferred prescription.

This study has certain limitations. First, although network consistency for HbA1c and FPG was favorable, substantial heterogeneity existed regarding sample sources, durations of disease, pharmacological treatments, supervision modalities, and the frequency and total volume of training. Second, the number of studies included for HOMA-IR was small. Although no statistically significant local inconsistency was detected, the node-splitting analysis suggested a borderline inconsistency signal for the comparison between MICT and HIIT-L. Together with the limited amount of evidence and wide credible intervals, these factors weaken the robustness of the HOMA-IR findings. The classification for HIIT was based solely on the duration of the high-intensity intervals. However, recovery duration, recovery mode, weekly frequency, total training volume, and energy expenditure might also influence the outcomes. Consequently, it is difficult to conclude that the observed differences were exclusively attributable to durations of intervals. Third, given the substantial inter-individual variability in exercise responsiveness among individuals with DM, more rigorously designed, dose-matched, head-to-head comparative studies are still required. Fourth, the risk of bias assessment discloses some concerns in several included trials, particularly regarding the randomization process, handling of missing data, and selective reporting, which may affect the stability of our findings. Fifth, publication bias and small-study effects could not be formally assessed because fewer than ten studies were available for each outcome network. Therefore, these potential sources of bias cannot be completely excluded. Sixth, several deviations from the PROSPERO registration occurred, including the use of RoB 2 alone, restriction to English-language publications, and the post-hoc inclusion of HOMA-IR as an exploratory secondary outcome. These deviations may have introduced language or selective-outcome bias and should be considered when interpreting the findings. Seventh, Winding et al. reported 32 arm-period observations from 26 unique completers because six participants initially assigned to the control condition were subsequently reallocated to an exercise group. Although the reported arm-level outcome data were retained, the resulting within-participant correlation could not be explicitly modeled using the available aggregate data. In the sensitivity analysis excluding this trial, the findings for FPG and HOMA-IR remained materially unchanged, whereas the HbA1c comparison between HIIT-L and MICT was no longer statistically significant. Therefore, this specific comparative finding should be interpreted cautiously.

Finally, the classification approach adopted in this research is not the only possible method. Nevertheless, based on existing evidence, this approach may facilitate the optimization of HIIT interventions and enhance their targeted efficacy, particularly for improving glycemic control in T2D.

## Conclusion

This Bayesian NMA compared different aerobic HIIT protocols, MICT, and CON for glycemic control in individuals with T2D. MICT, HIIT-S, and HIIT-L were all associated with reductions in HbA1c and FPG compared with CON. The estimated HbA1c reductions may also be clinically meaningful for many patients. In the primary analysis, HIIT-L produced a greater HbA1c reduction than MICT; however, this comparison was no longer statistically significant after excluding Winding et al., indicating that the apparent advantage was not fully robust. HIIT-L had the largest point estimate and highest ranking probability for FPG, but no statistically significant differences were observed among the active interventions. HIIT-S also improved glycemic control, and direct comparisons with HIIT-L remained inconclusive. Because each work interval is shorter, HIIT-S may be a practical alternative, although adherence, feasibility, and patient acceptability require direct evaluation. HOMA-IR findings remain exploratory because of the small evidence base, wide credible intervals, and sensitivity to model specification. Clinical selection among HIIT-L, HIIT-S, and MICT should be individualized according to medical status, baseline exercise capacity, cardiovascular risk, patient preference, and access to supervision. Future high-quality, volume-matched RCTs directly comparing HIIT-S and HIIT-L are needed to clarify the clinical utility of these protocols in T2D. 

## Supplementary Information

Supplementary Material 1.

Supplementary Material 2.

Supplementary Material 3.

Supplementary Material 4.

## Data Availability

All data generated or analysed during this study are included in this published article and its supplementary information files.

## Abbreviations

T2D: Type 2 diabetes
DM: Diabetes mellitus
HbA1c: Glycated hemoglobin
FPG: Fasting plasma glucose
CON: Non-exercise control
MICT: Moderate-intensity continuous training
HIIT: High-intensity interval training
HIIT-S: Short-interval HIIT
HIIT-L: Long-interval HIIT
NMA: Network meta-analysis
RCT: Randomized controlled trial
SIT: Sprint interval training
RST: Repeated sprint training
HOMA-IR: Homeostatic model assessment for insulin resistance
SD: Standard deviation
MD: Mean difference
CrI: Credible interval
SUCRA: Surface under the cumulative ranking curve
