# Supplementary material for: Comparative effects of long- and short-interval aerobic high-intensity interval training on glycemic control among individuals with type 2 diabetes: a network meta-analysis
Source: BMC Sports Sci Med Rehabil. 2026 Jul 27;18:408. doi: 10.1186/s13102-026-01921-4 (PMC13560113; doi:10.1186/s13102-026-01921-4)
Supplement: Supplementary file 1 [file 13102_2026_1921_MOESM1_ESM.docx]

**Additional file 1. PRISMA-NMA checklist**

Completed checklist for the manuscript: Comparative Effects of Long- and Short-Interval Aerobic High-Intensity Interval Training on Glycemic Control among Individuals with Type 2 Diabetes: A Network Meta-Analysis.

*Manuscript page references are based on the current 47-page manuscript file provided for revision. Items marked not assessed indicate that reporting bias or small-study effects were not formally evaluated because the number of studies within the outcome networks was limited.*

| **Section** | **Topic** | **Item** | **Checklist item** | **Reported on page(s)** |
| --- | --- | --- | --- | --- |
| TITLE | Title | 1 | Identify the report as a systematic review that incorporates network meta-analysis. | Page 1 |
| ABSTRACT | Structured summary | 2 | Summarize objectives, data sources, eligibility criteria, participants/interventions, appraisal, NMA methods, main results, limitations, conclusions, and registration when applicable. | Pages 1-3 |
| INTRODUCTION | Rationale | 3 | Explain why the review was needed in light of existing evidence. | Pages 4-7 |
| INTRODUCTION | Objectives | 4 | State the review questions using participants, interventions/comparators, outcomes, and study designs. | Page 7 |
| METHODS | Protocol and registration | 5 | Indicate protocol/registration information and where it can be accessed, or state if no registration was available. | Page 7 |
| METHODS | Eligibility criteria | 6 | Specify study and report characteristics used as eligibility criteria, with rationale where relevant. | Pages 8-9 |
| METHODS | Information sources | 7 | Describe databases and other information sources searched, with the date of the last search. | Pages 7-8 |
| METHODS | Search | 8 | Present the full search strategy or indicate where it is provided. | Pages 7-8; Supplementary Table S1 |
| METHODS | Study selection | 9 | Describe screening and selection procedures for included studies. | Pages 9 and 13-14; Figure 1 |
| METHODS | Data collection process | 10 | Describe data extraction procedures, including duplicate extraction or verification where relevant. | Pages 9-10 |
| METHODS | Data items | 11 | List the data items extracted and any assumptions or simplifications. | Page 10 |
| METHODS | Network geometry | S1 | Describe planned methods for examining and presenting the treatment network. | Pages 12-13 |
| METHODS | Risk of bias within studies | 12 | Describe methods for assessing risk of bias in individual studies. | Pages 10-11 |
| METHODS | Summary measures | 13 | State the principal summary measures used for treatment effects. | Pages 12-13 |
| METHODS | Synthesis methods | 14 | Describe methods of evidence synthesis, model choice, software, assumptions, rankings, and handling of heterogeneity. | Pages 12-13 |
| METHODS | Inconsistency assessment | S2 | Describe methods used to assess agreement between direct and indirect evidence. | Pages 12-13; Supplementary Table S2 |
| METHODS | Risk of bias across studies | 15 | Describe assessment of reporting bias or small-study effects, if performed. | Page 13 |
| METHODS | Additional analyses | 16 | Describe planned sensitivity, subgroup, or other additional analyses. | Pages 12-13 |
| RESULTS | Study selection | 17 | Report numbers screened, assessed, included, and excluded, with reasons. | Pages 13-14; Figure 1 |
| RESULTS | Study characteristics | 18 | Present characteristics of included studies. | Page 14; Table 2 |
| RESULTS | Risk of bias within studies | 19 | Present risk-of-bias results for individual studies. | Pages 14-15; Figure 2 |
| RESULTS | Network structure | S3 | Present the network of interventions/comparisons. | Pages 15-18; Figure 3 |
| RESULTS | Network geometry summary | S4 | Summarize the structure of the available evidence network and comparisons. | Pages 15-18 |
| RESULTS | Results of individual studies | 20 | Provide results for each study or comparison, including effect estimates where available. | Pages 15-18; Table 2 |
| RESULTS | Synthesis of results | 21 | Present meta-analysis/NMA results with effect estimates and uncertainty. | Pages 15-18; Tables 3-5; Figure 3; Supplementary Figures S1-S2 |
| RESULTS | Network meta-analysis results | S5 | Present relative effects and rankings from the NMA, with uncertainty and consistency information where available. | Pages 15-18; Tables 3-5; Figure 3; Supplementary Tables S2-S3; Supplementary Figures S1-S2 |
| RESULTS | Risk of bias across studies | 22 | Present results for reporting bias or small-study effects, if assessed. | Not assessed; see Methods, page 13. |
| RESULTS | Additional analyses | 23 | Report sensitivity or other additional analyses. | Page 18; Supplementary Table S3 |
| DISCUSSION | Summary of evidence | 24 | Summarize the main findings and the strength/relevance of the evidence. | Pages 19-23 |
| DISCUSSION | Limitations | 25 | Discuss limitations at study, outcome, and review levels. | Pages 19-23 |
| DISCUSSION | Conclusions | 26 | Provide overall interpretation and implications for practice/research. | Pages 23-24 |
| FUNDING | Funding | 27 | Describe funding sources and the role of funders. | Page 25 |

Checklist framework: PRISMA extension statement for systematic reviews incorporating network meta-analysis (PRISMA-NMA).
