# Supplementary material for: Comparative effects of long- and short-interval aerobic high-intensity interval training on glycemic control among individuals with type 2 diabetes: a network meta-analysis
Source: BMC Sports Sci Med Rehabil. 2026 Jul 27;18:408. doi: 10.1186/s13102-026-01921-4 (PMC13560113; doi:10.1186/s13102-026-01921-4)
Supplement: Supplementary file 2 [file 13102_2026_1921_MOESM2_ESM.docx]

**Supplementary Materials**

**Supplementary Table S1. Search strategy**

| **Database** | **Search date** | **Search strategy** | **Records identified** |
| --- | --- | --- | --- |
| PubMed | 24 October 2025 | #1 Diabetes Mellitus[MeSH Terms]; #2 diabetes[Title/Abstract] OR diabetic[Title/Abstract] OR diabetes mellitus[Title/Abstract] OR T2DM[Title/Abstract] OR T1DM[Title/Abstract] OR Prediabetes[Title/Abstract]; #3 High-Intensity Interval Training[MeSH Terms]; #4 Sprint Interval Training[Title/Abstract] OR high intensity interval training[Title/Abstract] OR HIIT[Title/Abstract] OR HIIE[Title/Abstract] OR high intensity interval exercise[Title/Abstract] OR sprint interval exercise[Title/Abstract] OR sprint interval training[Title/Abstract]; #5 (#1 OR #2) AND (#3 OR #4) | 532 |
| Embase | 24 October 2025 | #1 diabetes mellitus-related Emtree terms; #2 diabetes-related free-text terms; #3 high-intensity interval training-related Emtree terms; #4 HIIT/sprint interval training-related free-text terms; #5 (#1 OR #2) AND (#3 OR #4) | 1191 |
| Cochrane Library | 24 October 2025 | #1 MeSH descriptor: [Diabetes Mellitus] explode all trees; #2 diabetes OR diabetic OR diabetes mellitus OR T2DM OR T1DM OR Prediabetes:ti,ab,kw; #3 MeSH descriptor: [High-Intensity Interval Training] explode all trees; #4 Sprint Interval Training OR high intensity interval training OR HIIT OR HIIE OR high intensity interval exercise OR sprint interval exercise OR sprint interval training:ti,ab,kw; #5 (#1 OR #2) AND (#3 OR #4) | 628 |
| Web of Science | 24 October 2025 | #1 TS=(diabetes OR diabetic OR diabetes mellitus OR T2DM OR T1DM OR prediabetes); #2 TS=(Sprint Interval Training OR high intensity interval training OR HIIT OR HIIE OR high intensity interval exercise OR sprint interval exercise OR sprint interval training); #3 #1 AND #2 | 648 |

**Supplementary Table S2. Session structure and exercise-volume characteristics of the included interventions**

| **Study** | **Intervention arm** | **Classification** | **Exercise modality** | **Work interval duration** | **Repetitions** | **Work intensity** | **Recovery duration and mode** | **Recovery intensity** | **Work-to-recovery ratio** | **Warm-up/cool-down** | **High-intensity work per session, min** | **Estimated total session duration, min** | **Sessions per week** | **High-intensity work per week, min** | **Estimated total session time per week, min** | **Intervention duration** | **Notes** |
| --- | --- | --- | --- | --- | --- | --- | --- | --- | --- | --- | --- | --- | --- | --- | --- | --- | --- |
| Winding 2018 | HIIT-S | HIIT-S | Cycling | 1 min | 10 | 95% Wpeak | 1 min active recovery | 20% Wpeak | 1:1 | 5-min standardized warm-up | 10 | 25 | 3 | 30 | 75 | 11 weeks | Total session duration consistent with 75 min/week including warm-up. |
| Winding 2018 | MICT/END | MICT | Cycling | N/A | N/A | 50% Wpeak | N/A | N/A | N/A | 5-min standardized warm-up | N/A | 45 | 3 | N/A | 135 | 11 weeks | 40 min main continuous exercise plus warm-up. |
| Li 2022 | HIIT-S | HIIT-S | NR | Mixed intervals within 15-min HIIT block | NR | 80%-95% intensity | Active recovery within 15-min HIIT block | ~25% intensity | NR | 5-min warm-up + 5-min finishing activities | 8 | 25 | 5 | 40 | 125 | 12 weeks | Total session duration based on reported 5 + 15 + 5 min structure. |
| Li 2022 | MICT | MICT | NR | N/A | N/A | NR | N/A | N/A | N/A | NR | N/A | NR | NR | N/A | NR | 12 weeks | Detailed MICT dose not reported in the available extracted source. |
| Cassidy 2019 | HIIT-L | HIIT-L | NR | Progressive 2 min to 3 min 50 s | 5 | RPE 16-17 | 3 min recovery | NR | 0.67:1 to 1.28:1 | NR | 10-19.2 | approx. 22-34 | 3 | 30-57.5 | approx. 66-103 | 12 weeks | Estimated from work intervals plus 4-5 recovery periods; warm-up/cool-down NR. |
| Mitranun 2014 | HIIT-S | HIIT-S | NR | 1 min | 4-6 | 80%-85% VO2peak | 4 min active recovery | 50%-60% VO2peak | 1:4 | 30 min/session total reported | 4-6 | 30 | 3 | 12-18 | 90 | 12 weeks | Total session duration was reported. |
| Mitranun 2014 | MICT | MICT | NR | N/A | N/A | NR | N/A | N/A | N/A | NR | N/A | NR | NR | N/A | NR | 12 weeks | Detailed MICT dose not reported in the available extracted source. |
| Ahmad 2023 | HIIT-L low-volume | HIIT-L | NR | 4 min | 2 | 85%-90% peak HR | 3 min active recovery | 65%-75% peak HR | 4:3 | NR | 8 | >=11 | 3 | 24 | >=33 | 12 weeks | Minimum estimate excludes unreported warm-up/cool-down. |
| Ahmad 2023 | HIIT-L high-volume | HIIT-L | NR | 4 min | 4 | 85%-90% peak HR | 3 min active recovery | 65%-75% peak HR | 4:3 | NR | 16 | >=25 | 3 | 48 | >=75 | 12 weeks | Minimum estimate excludes unreported warm-up/cool-down. |
| Alizadeh 2025 | HIIT-S | HIIT-S | NR | 1 min | 7 | 90%-95% HRR | 2 min active recovery | 60%-70% HRR | 1:2 | NR | 7 | approx. 19-21 | 3 | 21 | approx. 57-63 | 8 weeks | Estimated from work and recovery intervals; warm-up/cool-down NR. |
| Alizadeh 2025 | MICT | MICT | NR | N/A | N/A | NR | N/A | N/A | N/A | NR | N/A | NR | NR | N/A | NR | 8 weeks | Detailed MICT dose not reported in the available extracted source. |
| Sabouri 2021 | HIIT-S | HIIT-S | NR | 1 min | 10 | 85%-90% HRmax | 1 min active recovery | NR | 1:1 | 5-min warm-up + 5-min cool-down at ~40% HRmax | 10 | approx. 29-30 | 3 | 30 | approx. 87-90 | 12 weeks | Range reflects uncertainty over final recovery interval. |
| Way 2020 | HIIT-L | HIIT-L | NR | 4 min | 1 | 90% VO2peak | N/A | N/A | N/A | 10-min warm-up + 5-min cool-down at 50% VO2peak | 4 | 19 | 3 | 12 | 57 | 12 weeks |  |
| Way 2020 | MICT | MICT | NR | N/A | N/A | NR | N/A | N/A | N/A | NR | N/A | NR | NR | N/A | NR | 12 weeks | Detailed MICT dose not reported in the available extracted source. |
| Mohammadi zadeh 2022 | HIIT-S | HIIT-S | NR | 1 min | 8-12 | 90%-100% HRmax | 2 min active recovery | 60%-70% HRmax | 1:2 | 4-5-min warm-up + 4-min cool-down | 8-12 | approx. 30-45 | 4 | 32-48 | approx. 120-180 | 12 weeks | Estimated from variable repetitions and recovery count. |
| Mohammadi zadeh 2022 | MICT | MICT | NR | N/A | N/A | NR | N/A | N/A | N/A | NR | N/A | NR | NR | N/A | NR | 12 weeks | Detailed MICT dose not reported in the available extracted source. |
| Kazemi 2024 | HIIT-S | HIIT-S | Cycling | 1 min | 10 | 90% HRmax | 1 min active recovery | 50 W | 1:1 | 3-min warm-up + 3-min cool-down at 50 W | 10 | approx. 25-26 | 3 | 30 | approx. 75-78 | 12 weeks | Range reflects uncertainty over final recovery interval. |
| Gentil 2023 | HIIT-L | HIIT-L | Walking/running | 2 min | 5 | 100% vVO2max | 2 min passive recovery | Passive recovery | 1:1 | 2-min warm-up + 2-min cool-down at 50% vVO2max | 10 | approx. 22-24 | 2 | 20 | approx. 44-48 | 8 weeks | Range reflects uncertainty over final recovery interval. |
| Gentil 2023 | HIIT-S | HIIT-S | Walking/running | 30 s | 20 | 100% vVO2max | 30 s passive recovery | Passive recovery | 1:1 | 2-min warm-up + 2-min cool-down at 50% vVO2max | 10 | approx. 23.5-24 | 2 | 20 | approx. 47-48 | 8 weeks | Range reflects uncertainty over final recovery interval. |
| Gentil 2023 | MICT | MICT | Walking/running | N/A | N/A | 70% vVO2max | N/A | N/A | N/A | 2-min warm-up + 2-min cool-down at 50% vVO2max | N/A | 18 | 2 | N/A | 36 | 8 weeks | Main continuous exercise was 14 min/session. |
| Niyazi 2024 | HIIT-L | HIIT-L | Walking/jogging | 4 min | 4 | 85%-95% HRmax | 3 min walking/jogging recovery | 50%-60% HRmax | 4:3 | 10-min warm-up at 50%-60% HRmax + 5-min cool-down | 16 | 40 | 3 | 48 | 120 | 12 weeks |  |
| Niyazi 2024 | MICT | MICT | Walking | N/A | N/A | 60%-70% HRmax | N/A | N/A | N/A | NR | N/A | 47 | 3 | N/A | 141 | 12 weeks |  |
| Findikoglu 2023 | HIIT-S | HIIT-S | Cycling | 1 min | 8-16 | 90% VO2peak | 120 s low-intensity recovery | 30% VO2peak | 1:2 | 5-min warm-up + 5-min cool-down at 20 W | 8-16 | approx. 32-58 | 3 | 24-48 | approx. 96-174 | 12 weeks | Estimated from variable repetitions and recovery count. |
| Findikoglu 2023 | MICT | MICT | NR | N/A | N/A | NR | N/A | N/A | N/A | NR | N/A | NR | NR | N/A | NR | 12 weeks | Detailed MICT dose not reported in the available extracted source. |
| Riahy 2024 | HIIT-L | HIIT-L | NR | 4 min | 4 | 85%-95% HRmax | 3 min active recovery | 50%-60% HRmax | 4:3 | 10-min warm-up at 50%-60% HRmax + 5-min cool-down | 16 | 40 | 3 | 48 | 120 | 12 weeks |  |
| Riahy 2024 | MICT | MICT | NR | N/A | N/A | NR | N/A | N/A | N/A | NR | N/A | NR | NR | N/A | NR | 12 weeks | Detailed MICT dose not reported in the available extracted source. |
| Abdi 2021 | HIIT-L | HIIT-L | Running | 4 min | 4 | 85%-95% HRmax | 3 min active recovery | 50%-60% HRmax | 4:3 | 10-min warm-up + 5-min cool-down at 40% HRmax | 16 | 40 | 3 | 48 | 120 | 12 weeks |  |
| Liu 2020 | HIIT-S | HIIT-S | Cycling | 50 s | 20 | 90% HRmax | 10 s passive recovery | 20% HRmax | 5:1 | NR | 16.7 | 20 | >=3 | >=50 | >=60 | 3 weeks | Total session duration was reported. |
| Liu 2020 | MICT | MICT | Continuous exercise | N/A | N/A | ~65% HRmax | N/A | N/A | N/A | NR | N/A | 50 | >=3 | N/A | >=150 | 3 weeks |  |

Note. Total session duration was calculated as the sum of reported warm-up, work intervals, recovery intervals, and cool-down. Where the number of recovery intervals or session components was unclear, values were reported as approximate ranges or minimum estimates. Estimated weekly values were calculated from session duration and reported training frequency. Energy expenditure and accumulated time above specific heart-rate or oxygen-uptake thresholds were not consistently reported and therefore could not be summarized across studies. NR indicates not reported; N/A indicates not applicable. HIIT, high-intensity interval training; MICT, moderate-intensity continuous training; HRmax, maximum heart rate; HRR, heart-rate reserve; VO₂peak, peak oxygen uptake; vVO₂max, velocity at maximal oxygen uptake; Wpeak, peak workload.

**Supplementary Table S3. Model fit, convergence diagnostics, and local inconsistency assessment**

**Part A. Model fit and convergence diagnostics**

| Outcome | Model | DIC | Residual deviance / data points | τ | 95% CrI for τ | PSRF | Convergence |
| --- | --- | --- | --- | --- | --- | --- | --- |
| HbA1c | Random-effects | 59.12 | 0.85 | 0.30 | 0.16 to 0.49 | 1.00 | Satisfactory |
| FPG | Random-effects | 44.77 | 0.82 | 0.15 | 0.01 to 0.48 | 1.00 | Satisfactory |
| HOMA-IR | Random-effects | 29.92 | 0.88 | 0.48 | 0.03 to 1.22 | 1.00 | Satisfactory |

**Part B. Node-splitting analysis of local inconsistency**

| Outcome | Comparison | Direct estimate, MD (95% CrI) | Indirect estimate, MD (95% CrI) | Network estimate, MD (95% CrI) | P value | Interpretation |
| --- | --- | --- | --- | --- | --- | --- |
| HbA1c | CON vs MICT | −0.47 (−0.85, −0.09) | −0.14 (−1.30, 1.00) | −0.47 (−0.77, −0.16) | 0.585 | No significant inconsistency |
| HbA1c | CON vs HIIT-S | −0.39 (−0.77, −0.01) | −0.87 (−1.70, −0.03) | −0.50 (−0.84, −0.18) | 0.290 | No significant inconsistency |
| HbA1c | CON vs HIIT-L | −0.90 (−1.20, −0.60) | −1.30 (−2.90, 0.23) | −0.84 (−1.10, −0.55) | 0.595 | No significant inconsistency |
| HbA1c | MICT vs HIIT-S | −0.16 (−0.50, 0.19) | 0.29 (−0.85, 1.50) | −0.04 (−0.37, 0.29) | 0.458 | No significant inconsistency |
| HbA1c | MICT vs HIIT-L | −0.12 (−0.65, 0.39) | −0.68 (−1.30, −0.11) | −0.38 (−0.75, −0.01) | 0.149 | No significant inconsistency |
| HbA1c | HIIT-S vs HIIT-L | −0.92 (−2.60, 0.77) | −0.31 (−0.74, 0.14) | −0.34 (−0.76, 0.08) | 0.488 | No significant inconsistency |
| FPG | CON vs MICT | −0.69 (−1.10, −0.14) | −1.40 (−2.90, 0.11) | −0.77 (−1.10, −0.43) | 0.390 | No significant inconsistency |
| FPG | CON vs HIIT-S | −0.78 (−1.20, −0.34) | −1.30 (−2.50, −0.14) | −0.86 (−1.20, −0.52) | 0.381 | No significant inconsistency |
| FPG | CON vs HIIT-L | −1.20 (−1.70, −0.74) | −0.72 (−3.00, 1.40) | −1.10 (−1.50, −0.71) | 0.684 | No significant inconsistency |
| FPG | MICT vs HIIT-S | −0.13 (−0.47, 0.20) | −0.30 (−2.10, 1.50) | −0.09 (−0.40, 0.22) | 0.854 | No significant inconsistency |
| FPG | MICT vs HIIT-L | 0.11 (−0.71, 0.94) | −0.59 (−1.40, 0.05) | −0.36 (−0.84, 0.14) | 0.171 | No significant inconsistency |
| FPG | HIIT-S vs HIIT-L | 0.00 (−2.40, 2.60) | −0.29 (−0.82, 0.27) | −0.27 (−0.76, 0.25) | 0.799 | No significant inconsistency |
| HOMA-IR | MICT vs HIIT-S | −0.71 (−1.80, 0.45) | 0.13 (−2.10, 2.50) | −0.30 (−1.30, 0.65) | 0.473 | No significant inconsistency |
| HOMA-IR | MICT vs HIIT-L | −0.12 (−0.98, 0.83) | −2.00 (−3.70, −0.12) | −0.41 (−1.50, 0.35) | 0.066 | Borderline inconsistency signal; interpret cautiously |

Note. MD, mean difference; CrI, credible interval; DIC, deviance information criterion; PSRF, potential scale reduction factor. A P value <0.05 was considered indicative of statistically significant local inconsistency. Estimates are rounded to two decimal places.

**Supplementary Table S4. Sensitivity analysis using fixed-effect models**

| Outcome | Comparison | Random-effects model | Fixed-effect model | Consistency | Interpretation |
| --- | --- | --- | --- | --- | --- |
| HbA1c | MICT vs CON | −0.47 (−0.77, −0.17) | −0.41 (−0.59, −0.23) | Consistent | Robust |
| HbA1c | HIIT-S vs CON | −0.50 (−0.84, −0.18) | −0.46 (−0.65, −0.26) | Consistent | Robust |
| HbA1c | HIIT-L vs CON | −0.85 (−1.16, −0.55) | −0.78 (−0.93, −0.64) | Consistent | Robust |
| HbA1c | HIIT-L vs MICT | −0.38 (−0.76, −0.01) | −0.37 (−0.59, −0.16) | Consistent | Robust in model comparison, but sensitive to Winding exclusion |
| HbA1c | HIIT-L vs HIIT-S | −0.34 (−0.76, 0.08) | −0.33 (−0.56, −0.09) | Not consistent | Sensitive to model specification |
| FPG | MICT vs CON | −0.77 (−1.10, −0.43) | −0.77 (−1.06, −0.48) | Consistent | Robust |
| FPG | HIIT-S vs CON | −0.85 (−1.21, −0.52) | −0.85 (−1.14, −0.57) | Consistent | Robust |
| FPG | HIIT-L vs CON | −1.12 (−1.54, −0.70) | −1.13 (−1.47, −0.78) | Consistent | Robust |
| FPG | Comparisons among active interventions | No statistically significant differences | No statistically significant differences | Consistent | Conclusions unchanged |
| HOMA-IR | Exercise interventions vs CON | Only HIIT-L was statistically significant | MICT, HIIT-S, and HIIT-L were statistically significant | Not consistent | Sensitive to model specification |
| HOMA-IR | MICT vs HIIT-L: local inconsistency | No statistically significant inconsistency (P = 0.066) | Statistically significant local inconsistency | Not consistent | Interpret cautiously |

Note. The random-effects model was retained as the primary model because of clinical and methodological heterogeneity across trials. For HOMA-IR, the table summarizes the statistical-significance and inconsistency patterns reported in the manuscript.

**Supplementary Table S5. Sensitivity analysis excluding Winding et al.**

| Outcome | Comparison | Main analysis, MD (95% CrI) | Excluding Winding et al., MD (95% CrI) | Main rank | Rank after exclusion | Interpretation |
| --- | --- | --- | --- | --- | --- | --- |
| HbA1c | MICT vs CON | −0.47 (−0.77, −0.17) | −0.51 (−0.84, −0.19) | 3 | 3 | Materially unchanged |
| HbA1c | HIIT-S vs CON | −0.50 (−0.84, −0.18) | −0.54 (−0.92, −0.19) | 2 | 2 | Materially unchanged |
| HbA1c | HIIT-L vs CON | −0.85 (−1.16, −0.55) | −0.86 (−1.18, −0.55) | 1 | 1 | Materially unchanged |
| HbA1c | HIIT-L vs MICT | −0.38 (−0.76, −0.01) | −0.35 (−0.74, 0.05) | — | — | No longer statistically significant |
| FPG | MICT vs CON | −0.77 (−1.10, −0.43) | −0.76 (−1.11, −0.42) | 3 | 3 | Materially unchanged |
| FPG | HIIT-S vs CON | −0.85 (−1.21, −0.52) | −0.84 (−1.20, −0.50) | 2 | 2 | Materially unchanged |
| FPG | HIIT-L vs CON | −1.12 (−1.54, −0.70) | −1.12 (−1.55, −0.68) | 1 | 1 | Materially unchanged |
| HOMA-IR | MICT vs CON | −0.42 (−1.09, 0.38) | −0.40 (−1.07, 0.37) | 3 | 3 | Materially unchanged |
| HOMA-IR | HIIT-S vs CON | −0.71 (−1.61, 0.25) | −0.70 (−1.61, 0.27) | 2 | 2 | Materially unchanged |
| HOMA-IR | HIIT-L vs CON | −0.84 (−1.74, −0.06) | −0.85 (−1.71, −0.07) | 1 | 1 | Materially unchanged |

Note. MD, mean difference; CrI, credible interval. Treatment ranks refer to active exercise interventions, with rank 1 indicating the highest SUCRA ranking. Values are rounded to two decimal places.
